# Predicting Sasang Constitution Using Body-Shape Information

**DOI:** 10.1155/2012/398759

**Published:** 2012-06-25

**Authors:** Eunsu Jang, Jun-Hyeong Do, HeeJeong Jin, KiHyun Park, Boncho Ku, Siwoo Lee, Jong Yeol Kim

**Affiliations:** Division of Constitutional Medicine/Diagnosis Research Group, Korea Institute of Oriental Medicine, 1672 Yuseongdae-ro, Yuseong-gu, Daejeon 305-811, Republic of Korea

## Abstract

*Objectives*. Body measurement plays a pivotal role not only in the diagnosis of disease but also in the classification of typology. Sasang constitutional medicine, which is one of the forms of Traditional Korean Medicine, is considered to be strongly associated with body shape. We attempted to determine whether a Sasang constitutional analytic tool based on body shape information (SCAT-B) could predict Sasang constitution (SC). *Methods*. After surveying 23 Oriental medical clinics, 2,677 subjects were recruited and body shape information was collected. The SCAT-Bs for males and females were developed using multinomial logistic regression. Stepwise forward-variable selection was applied using the score statistic and Wald's test. *Results*. The predictive rates of the SCAT-B for Tae-eumin (TE), Soeumin (SE), and Soyangin (SY) types in males and females were 80.2%, 56.9%, and 37.7% (males) and 69.3%, 38.9%, and 50.0% (females) in the training set and were 74%, 70.1%, and 35% (males), and 67.4%, 66.3%, and 53.7% (females) in the test set, respectively. Higher constitutional probability scores showed a trend for association with higher predictability. *Conclusions*. This study shows that the Sasang constitutional analytic tool, which is based on body shape information, may be relatively highly predictive of TE type but may be less predictive when used for SY type.

## 1. Introduction

Larger body measurement indices are known to be associated with disease; thus, specific ranges have been established to enable disease prediction. A body mass index (BMI) greater than 30 is a well-known risk factor for metabolic syndrome [1]. A waist circumference (WC) of greater than 102 cm in males and 88 cm in females has been recognized as an effective predictor of heart disease [2]. Waist-to-hip ratio has consistently been reported to correlate with cardiovascular disease. Furthermore, the ratio of specific body parts has also been reported to be associated with mental capacity [3, 4]. Therefore, information on body measurements and specific ratios between these indices can be important predictors of disease.

Sasang constitutional medicine (SCM), which is one of the forms of Traditional Korean Medicine (TKM), classifies people into four different constitution types: Taeyangin (TY), Soyangin (SY), Tae-eumin (TE), and Soeumin (SE) [5]. Because SCM experts use different prescriptions to treat the same disease in different constitution types or use an identical method to differentiate diseases presenting in different constitutions, they attempt to accurately diagnose the patient's constitution in advance [6]. To determine a patient's constitution, experts observe body shape, face, voice, temperament, physiological and pathological symptoms, and disease characteristics. Among these factors, body shape plays a crucial role in predicting Sasang constitution (SC) types [7, 8]. For example, it is known that waste discharging function can be hypoactive in SY types, whereas a small hip circumference and a large WC associates with TE types [9, 10]. Height, weight, BMI, and eight circumference measurements are widely known to represent the body's constitutional characteristics; thus, these measurements have been utilized extensively in the field of Korean SCM [11, 12]. We have studied the reliability of body measurements [13] and suggested that those measurements could be reliable if obtained with a standard operating procedure (SOP) [14]. 

In this study, we developed a Sasang constitutional analytic tool based on body shape information (SCAT-B). We attempted to determine whether it could predict SC type to aid SCM doctors in TKM clinics. 

## 2. Materials and Methods

### 2.1. Subject Recruitment and SCAT-B Data Collection

After surveying 23 TKM clinics, 2,488 subjects ranging in age from 10–80 years old were recruited from November 2007 to July 2010. The subject's SC types were confirmed by experts. To ensure that the diagnosis was correct, we strictly controlled the quality of the practitioners, who had more than five years of experience in clinical practice. A more detailed procedure for determining SC types has been presented previously [15]. 

We collected eight circumference measurements of body shape: forehead circumference (FC), neck circumference (NC), axillary circumference (AC), chest circumference (CC), rib circumference (RC), waist circumference (WC), pelvic circumference (PC), and hip circumference (HC). We also measured the height and weight of each subject based on the SOP developed for the Korea Constitution Multicenter Study [12, 14]. 

We had to exclude 65 TY-type subjects due to the small sample size; 67 subjects who were younger than 15 years old were also excluded because they had a premature body shape. In addition, 142 subjects were excluded due to data extraction errors, and the presence of abnormal outliers and influential samples in their data sets. The general characteristics of the 2,214 subjects who were assigned to the training set are described in Table 1. This study was approved by the Institutional Review Board at the KIOM (I-0910/02-001).

### 2.2. Statistical Modeling for the SCAT-B

The 8 circumference measurements and the ratios of all possible pairs and the weight, height, and BMI were used to capture body shape characteristics.

We performed all analyses using SPSS 17.0 statistical software (SPSS Inc., Chicago, IL). The SCAT-Bs were developed separately for males and females using multinomial logistic regression (MLR). Selected variables based on SC were compared using one-way ANOVA, and Scheffe's test was conducted when the variables differed significantly (*P* < .05). Stepwise forward-variable selection utilizing the Wald's test was applied [16, 17], and the score statistic was used to directly calculate the probability of the MLR. SC types were predicted using the maximum value from three constitutional probability scores. The Cohen Kappa coefficient was used to determine the predictive power. 

Because the candidate variables showed age-specific trends, we calculated moving averages and standard deviations within a range of ±5 years. Age was used as a baseline covariant for the adjustment. After constructing the SCAT-B with the training set, we confirmed the predictability of this information in the test set. The selected variables and estimated parameters created by the MLR are summarized in Supplementary Tables S1 and S2 available online at doi:10.1155/2012/398759.

### 2.3. Predictability of SCAT-B

To determine whether the SCAT-B could aid local clinicians, we recruited 209 males and 254 females from 8 TKM clinics from February 2011 to December 2011. We collected the same body shape information from the subjects based on the same SOP [15].

The general characteristics of these males and females are described in Table 1. The predictability and the Kappa coefficients between the SCAT-B and the expert diagnosis were analyzed based on the algorithm derived from the training set. All analyses were performed using the method described in 2.2.

## 3. Results

### 3.1. Selected Candidate Variables of the SCAT-B

 The BMI, weight, NC, HC/FC, WC/NC, and CC/AC variables in males and BMI, weight, AC, CC, HC/NC, and CC/AC variables in females were selected in the SCAT-B models using MLR, and stepwise forward-selection from candidate variables. Most selected variables from TE types were significantly larger than in other types and were followed in size by those of SY and then SE types. The detailed characteristics of the variables that were used in the MLR are presented in Table 2.

### 3.2. Predictability of the SCAT-B in the Training Set

The predictive rates of the SCAT-B for TE, SE, and SY males and females were 80.2%, 56.9%, and 37.7% (males) and 69.3%, 38.9%, and 50.0% (females), respectively. This result revealed that predictability was relatively high for TE but was comparatively low for SE and SY. 

The total weighted predictive rates were 61.3% and 54.2% for males and females, respectively. This result revealed that predictability was higher for males than for females. 

The Cohen Kappa coefficients were 0.387 and 0.298 for males and females, respectively. The predictability and Kappa coefficients of the SCAT-B are shown in Table 3.

**Figure 1:**
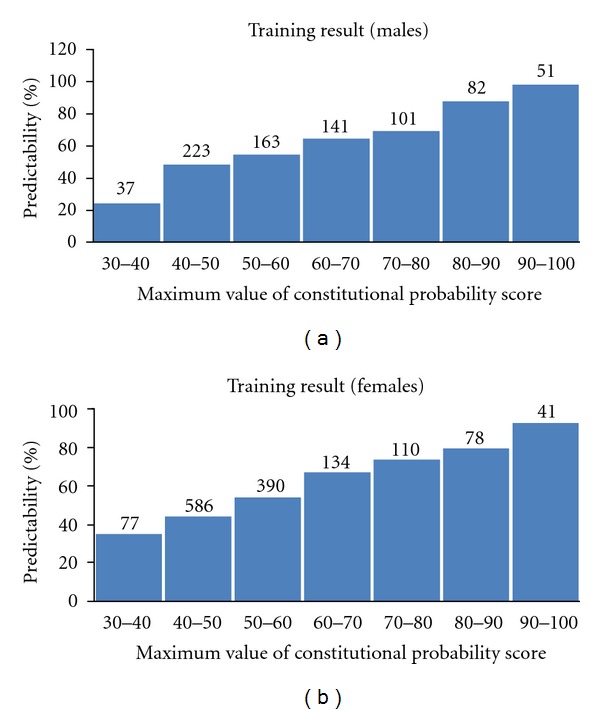
Predictability of the SCAT-B based on the probability score in the training set.

These data show an increasing trend of predictability as the maximum constitutional probability score increases. This finding indicated that the higher the probability score, the more accurate the SCAT-B. The detailed results based on the maximum value of the probability score are presented in Figure 1. 

The predictability of the SCAT-B was increased when the cut-off value of the constitutional probability score was over 40. In this case, the predictability of the SCAT-B became 63.1% (Kappa = 0.411) and 55.3% (Kappa = 0.314) for males and females, respectively. If the cut-off value of the constitutional probability score over 50, it became 69.1% (Kappa = 0.470) and 64% (Kappa = 0.429) for males and females, respectively.

### 3.3. Predictability of the SCAT-B in the Test Set

The predictive rates of the SCAT-B for TE, SE, and SY males and females in the test set were 60.5%, 81.5%, and 30.3% (males), and 66.4%, 72%, and 43.4% (females), respectively. This result revealed that predictability was relatively high for TE but was comparatively low for SY. 

The total weighted predictive rates were 65.1% for males and 63.4% for females. Thus, predictability for males was higher than for females.

The Kappa coefficient was 0.458 for males and 0.449 for females. The predictability and Kappa coefficient of the SCAT-B in the test set are shown in Table 4. The training and test data sets were similar, with predictive rates being largest in TE types, followed by the SY and SE types. 

**Figure 2:**
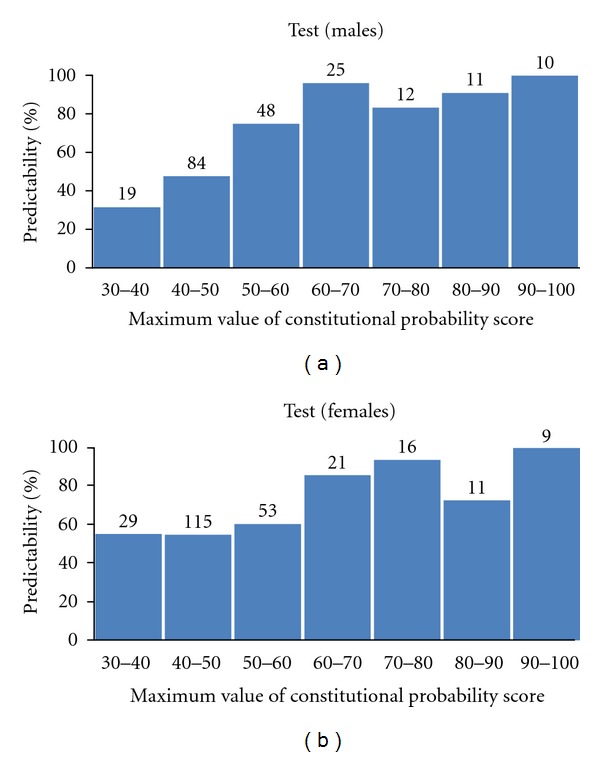
Predictability of the SCAT-B based on the probability score in the test set.

These data also show an increasing trend of predictability as the maximum constitutional probability score increases. These data also indicated that higher probability scores correlated with more accurate SCAT-B. The detailed results based on the maximum value of the probability score are shown in Figure 2.

If a cut-off value for the constitutional probability score was set to greater than 40, the predictability of the SCAT-B was 68.4% (Kappa = 0.501) for males and 64.4% (Kappa = 0.464) for females. If a cut-off of greater than 50 was used, the predictability became 84.9% (Kappa = 0.731) for males and 74.5% (Kappa = 0.589) for females. 

## 4. Discussion and Conclusion 

By developing a Sasang constitutional analytic model using body shape information (SCAT-B) from multiple nationwide centers, we attempted to determine whether the body measurement index could predict SC types accurately and whether this SCAT-B might assist local experts.

The MLR and stepwise forward-selection chose several influential variables from candidates for the SCAT-B models. That analysis allowed the SCAT-Bs to contain both BMI and weight variables and some of the 8 different circumferences or their ratios for males and females. While significant variables including high BMI and weight appeared to indicate characteristics of TE, other significant variables including low BMI and a low CC/AC ratio showed characteristics of SE types. This finding is in line with “longevity and life preservation in oriental medicine,” which states that the TE type is large with a relatively well-developed abdomen whereas the SE type is of small with a relatively undeveloped chest [10]. However, the SCAT-Bs did not reveal specific variables that represented SY characteristics.

The predictability of the TE was relatively high (80.2%), but the predictability for SY was comparatively low (37.7%) in males. Thus, the model is effective for TE type males but has some difficulty in classifying SY types. In females, while the predictability for TE was also relatively high (69.3%), the predictabilities for SE and SY were comparatively low (38.9% and 50%, resp.). These data revealed that the model was effective for TE type females but had some difficulty when classifying SE and SY types. Thus, it appears that the two SCAT-Bs have a similarly high capacity to predict TE type but may be less effective when predicting SE and SY types.

The test results demonstrated very similar trends to those of the training set in males, in that the predictability for TE was highest (74%) and SY predictability was lowest (35.7%). However, in females, although the test data showed a similar ability to predict TE (67.4%) type as the training data, the type with the lowest prediction rate changed from SE to SY (53.7%). This result revealed that gender differences in body shape appeared to influence constitutional predictability differently in males and females. In view of these results, we stress that SCAT-Bs show relatively high predictability for TE but comparatively low predictability for SY.

Some diagnoses of relatively low predictability are inevitable in many classification models that place a priority on overall high predictability. In this study, MLR analysis developed a linear predictor function for the overall highly predictive rate by constructing a score from a set of weights that are linearly combined with the explanatory variables of a given observation. For this reason, the predictive rates for the SY type in males and the SE type in females appeared relatively low in our model whereas the overall predictive rate for each gender may be reasonable as a result.

The predictability of the SCAT-B in males (61.3%) and females (54.2%) was similar to that of the Sasang Constitutional Classification Questionnaire (QSCC) [18], which is a well-known tool used in diagnosing SC types. The predictability of this questionnaire is known to vary from 51% to 70% [19, 20]. The predictability of each type when using SCAT-B compared to when using QSCC. Whereas the SCAT-B showed high predictability for TE and low predictability for SY, the QSCC showed low predictability for TE. Thus, the predictability of each type when using SCAT-B may be different to when using QSCC. However, QSCC has been criticized due to its subjective qualitative questions that depend upon one's own viewpoint. SCAT-B information could be much more meaningful because the data are derived strictly from quantitative body shape information.

 SCAT-B predictive trends using body shape were slightly different than the trends obtained from voice diagnosis. Using the vocal feature, Sasang constitutional diagnosis revealed a relatively high accuracy in predicting SE type (from 50 to 58%) and relatively low prediction of SY type (from 40% to 49%) [21]. These data suggest that individual constitutional diagnostic methods show different predictions depending on the SC. Therefore, if other SC factors, such as voice, face, characteristics, and symptoms, are integrated with SCAT-B, then the predictive capability of the integrated diagnostic model would be increased.

However, the SCAT-B did not perform exceptionally well [22] because the Cohen Kappa coefficients varied from 0.298 and 0.387 in the training set to 0.458 and 0.449 in the test set, which represents just fair agreement. 

The insufficient predictability of the SCAT-B may be explained in several ways. First, only body shape information, which is only one SCM factor, was used to develop the model although there are other factors that can be used to predict SC types, such as face, voice, and temperament. If we combine other quantitative factors with body shape, it may be possible to improve the predictive power of SCAT-B. Second, some of the variables extracted from body shape may not show clear differences between SC types (S1, S2). Thus, clear boundaries were not defined for SC types, and it remains difficult to fully describe SC characteristics. Third, the predictability of the SCAT-B was much lower for SY type subjects. Therefore, the use of this measure may be responsible for the insufficient predictability of the SCAT-B, although it is reported in “Longevity and life preservation in oriental medicine” that the SY type is easily predicted [10]. Because SY-type characteristics have been known to influence temperament, experts would require information on the patient's temperament in cases where the SCAT-B predicts that the patient is of SY type. 

This study revealed that higher constitutional probability values were associated with higher predictability in both training and test results datasets. Thus, if SC was predicted using SCAT-Bs based on constitutional probability scores of greater than 50, the predictability was almost 10% higher than the original result obtained in the training and test sets and the Kappa coefficients increased to over 0.4 in the training set and more than 0.5 in the test set, indicating substantial agreement [22]. These data indicated that the higher probability score, the more accurate the prediction; thus, by providing a probability score, SCAT-B could assist experts make diagnoses with stratified levels of confidence.

We compensated for age differences by using statistical methods for age-specific trends in the body measurement variables. However, we did not adjust for other factors that could affect body shape, such as physical activity and food intake. These factors may affect the predictability of the SCAT-B.

In future analyses, it may be necessary to integrate other factors, such as voice, face, and temperament, to improve predictability, and this integration will also be required to determine the predictability of the TY type, thereby completing the four-type diagnosis. 

In this study, we developed a diagnostic model for SC types using body shape information and evaluated the predictability of the model. We believe the use of the SCAT-B for both males and females may assist with predicting TE type but may be less effective when used for prediction of SE and SY types.

## Supplementary Material

These are the results of multinomial logistic regression based on stepwise forward variable selection using Wald's test in both males and females. The each reference category is set to TE type, which is used as the basis for comparison to SE and SY types. B represents estimated beta coefficients for selected variables, which can be interpreted as the magnitude to classify SC types. Furthermore, p-values corresponding to each variable indicate the statistical significance at *α*=0.05, which are derived from Wald statistics to test the null hypothesis *H*_0_:**β*_i_*=0,*i*=1,…,*k*, where k is the number of selected variables. The result of goodness of fit test for the model was summarized by log likelihood and *χ* ^2^-statistics. Finally, Nagelkerke's *R^2^* indicates the power of explanation to predict categorical responses for the model derived from selected explanatory variables.

## Figures and Tables

**Table 1:** General characteristics of the subjects.

	Gender	SC types (*N*)	Age (year)	Height (cm)	Weight (kg)
Training set	Males	TE (358)	48.58 ± 14.96	170.32 ± 6.02	75.28 ± 10.29
		SE (188)	44.53 ± 15.4	169.64 ± 6.22	62.38 ± 7.39
		SY (252)	49.19 ± 15.12	169.15 ± 6.16	67.21 ± 8.69
	Females	TE (521)	49.91 ± 15.07	157.6 ± 5.93	62.56 ± 8.69
		SE (375)	45.56 ± 15.32	158.08 ± 5.64	52.72 ± 6.34
		SY (520)	46.2 ± 14.09	157.16 ± 5.91	55.27 ± 6.83

Test set	Males	TE (100)	37.53 ± 16.46	173.51 ± 6.34	75.81 ± 10.17
		SE (67)	34.9 ± 15.13	172.67 ± 6.71	62.79 ± 6.56
		SY (42)	34.93 ± 12.4	173.36 ± 5.61	66.37 ± 6.97
	Females	TE (95)	44.97 ± 17.96	160.01 ± 6.56	62.05 ± 8.38
		SE (92)	40.39 ± 14.56	159.8 ± 6.55	50.08 ± 5.96
		SY (67)	38.67 ± 15.46	159.67 ± 6.17	52.31 ± 5.78

All values are of the M ± SD; M: mean; SD: standard deviation.

**Table 2:** The Characteristics of selected variables according to gender and Sasang constitution in the training set.

		Sasang constitution			*P* value	Scheffe's test
	Variables	TE	SE	SY		
Males	Number	358	188	252		
	BMI (kg/m^2^)	25.89 ± 2.78	21.67 ± 2.26	23.46 ± 2.48	<.001	TE > SY > SE
	Weight (Kg)	75.28 ± 10.29	62.38 ± 7.39	67.21 ± 8.69	<.001	TE > SY > SE
	NC (cm)	39.79 ± 2.47	36.53 ± 1.98	37.86 ± 2.31	<.001	TE > SY > SE
	HC/FC	1.67 ± 0.1	1.6 ± 0.09	1.63 ± 0.09	<.001	TE > SY > SE
	WC/NC	2.32 ± 0.16	2.22 ± 0.17	2.26 ± 0.15	<.001	TE > SY > SE
	CC/AC	0.98 ± 0.03	0.97 ± 0.03	0.98 ± 0.03	<.001	TE, SY > SE

Females	Number	521	375	520		
	BMI (kg/m^2^)	25.19 ± 3.23	21.12 ± 2.56	22.4 ± 2.7	<.001	TE > SY > SE
	Weight (Kg)	62.56 ± 8.69	52.72 ± 6.34	55.27 ± 6.83	<.001	TE > SY > SE
	AC (cm)	91.25 ± 6.09	83.77 ± 5.6	85.67 ± 5.61	<.001	TE > SY > SE
	CC (cm)	94.96 ± 7.33	85.45 ± 6.51	88.2 ± 7.02	<.001	TE > SY > SE
	HC/NC	2.79 ± 0.19	2.81 ± 0.17	2.78 ± 0.18	0.128	
	CC/AC	1.04 ± 0.04	1.02 ± 0.04	1.03 ± 0.04	<.001	TE > SY > SE

All values are of the M ± SD otherwise indicated; M: mean; SD: standard deviation.

**Table 3:** Predictability and Kappa coefficients of the SCAT-B in the training set.

Gender	Expert diagnosis	SCAT-B diagnosis (*N*, %)				Predictability (%)	Kappa
		TE	SE	SY	Total
Males	TE	287 (80.2)	18 (5)	53 (14.8)	358 (100)	489 (61.3)	.387
	SE	33 (17.6)	107 (56.9)	48 (25.5)	188 (100)		
	SY	102 (40.5)	55 (21.8)	95 (37.7)	252 (100)		

Females	TE	361 (69.3)	21 (4)	139 (26.7)	521 (100)	767 (54.2)	.298
	SE	52 (13.9)	146 (38.9)	177 (47.2)	375 (100)		
	SY	149 (28.7)	111 (21.3)	260 (50)	520 (100)		

**Table 4:** Predictability and Kappa coefficient of the SCAT-B in the test set.

Gender	Expert diagnosis	SCAT-B diagnosis (*N*, %)				Predictability (%)	Kappa
		TE	SE	SY	Total
Males	TE	74 (74)	4 (4)	22 (22)	100 (100)	136 (65.1)	.458
	SE	3 (4.5)	47 (70.1)	17 (25.4)	67 (100)		
	SY	11 (26.2)	16 (38.1)	15 (35.7)	42 (100)		

Females	TE	64 (67.4)	17 (17.9)	14 (14.7)	95 (100)	153 (63.4)	.449
	SE	3 (3.3)	61 (66.3)	28 (30.4)	92 (100)		
	SY	6 (9)	25 (37.3)	36 (53.7)	67 (100)		
