# Supplementary material for: Predicting Sasang Constitution Using Body-Shape Information
Source: Evid Based Complement Alternat Med. 2012 Jun 25;2012:398759. doi: 10.1155/2012/398759 (PMC3389671; doi:10.1155/2012/398759)
Supplement: Supplementary file 1 — These are the results of multinomial logistic regression based on stepwise forward variable selection using Wald's test in both males and females. The each reference category is set to TE type, which is used as the basis for comparison to SE and SY types. B represents estimated beta coefficients for selected variables, which can be interpreted as the magnitude to classify SC types. Furthermore, p-values corresponding to each variable indicate the statistical significance at α=0.05, which are derived from Wald statistics to test the null hypothesis H 0:βi=0,i=1,…,k, where k is the number of selected variables. The result of goodness of fit test for the model was summarized by log likelihood and χ 2-statistics. Finally, Nagelkerke's R2 indicates the power of explanation to predict categorical responses for the model derived from selected explanatory variables. [file 398759.f1.docx]

**Supplementary Data**

Table S1. Selected Variables and Estimated SCAT-B Parameters in Males

*Model
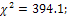

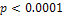
, -2 log likelihood=1304.3, pseudo
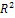
(Nagelkerke)=0.442

| SC type |  | †B | SE | Wald | df | P value |
| --- | --- | --- | --- | --- | --- | --- |
| SE | Intercept | -0.348 | 0.377 | 0.854 | 1 | .355 |
|  | Age | -0.011 | 0.008 | 2.099 | 1 | .147 |
|  | Weight | -0.594 | 0.314 | 3.570 | 1 | .059 |
|  | BMI | -1.141 | 0.299 | 14.581 | 1 | <.0001 |
|  | HC/FC | 0.438 | 0.147 | 8.872 | 1 | .003 |
|  | WC/NC | -0.337 | 0.168 | 4.046 | 1 | .044 |
|  | CC/AC | -0.006 | 0.124 | 0.002 | 1 | .960 |
|  | RC/AC | -0.242 | 0.131 | 3.415 | 1 | .065 |
|  | NC | -0.931 | 0.213 | 19.062 | 1 | <.001 |
| SY | Intercept | -0.399 | 0.321 | 1.543 | 1 | .214 |
|  | AGE | 0.008 | 0.006 | 1.583 | 1 | .208 |
|  | Weight | -0.286 | 0.241 | 1.408 | 1 | .235 |
|  | BMI | -0.553 | 0.232 | 5.682 | 1 | .017 |
|  | HC/FC | 0.308 | 0.119 | 6.706 | 1 | .010 |
|  | WC/NC | -0.390 | 0.14 | 7.75 | 1 | .005 |
|  | CC/AC | 0.364 | 0.104 | 12.353 | 1 | <.001 |
|  | RC/AC | -0.249 | 0.11 | 5.159 | 1 | .023 |
|  | NC | -0.674 | 0.175 | 14.793 | 1 | <.001 |

*References category: TE type

†B: estimated coefficient, S.E: standard error

Table S2. Selected Variables and Estimated SCAT-B Parameters in Females

*Model
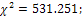

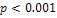
, -2 log likelihood=2548.9, pseudo
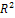
(Nagelkerke)=0.353

| SC type |  | †B | SE | Wald | df | P value |
| --- | --- | --- | --- | --- | --- | --- |
| SE | Intercept | 0.367 | 0.276 | 1.77 | 1 | .183 |
|  | Age | -0.018 | 0.005 | 10.880 | 1 | <.001 |
|  | Weight | -0.211 | 0.206 | 1.046 | 1 | .306 |
|  | BMI | -1.092 | 0.209 | 27.306 | 1 | <.001 |
|  | AC | -1.794 | 2.078 | 0.745 | 1 | .388 |
|  | CC/AC | -0.908 | 1.101 | 0.681 | 1 | .409 |
|  | HC/NC | 0.339 | 0.082 | 17.133 | 1 | <.001 |
|  | CC | 1.219 | 2.524 | 0.233 | 1 | .629 |
| SY | Intercept | 0.940 | 0.238 | 15.602 | 1 | <.001 |
|  | Age | -0.014 | 0.005 | 9.246 | 1 | .002 |
|  | Weight | -0.514 | 0.173 | 8.853 | 1 | .003 |
|  | BMI | -0.242 | 0.169 | 2.044 | 1 | .153 |
|  | AC | -6.844 | 1.829 | 13.998 | 1 | <.001 |
|  | CC/AC | -3.411 | 0.97 | 12.369 | 1 | <.001 |
|  | HC/NC | 0.127 | 0.069 | 3.345 | 1 | .067 |
|  | CC | 7.572 | 2.202 | 11.824 | 1 | <.001 |

*References category: TE type

†B: estimated coefficient, S.E: standard error
